# Diagnosis challenges in inception cohorts in axial spondyloarthritis: the case of the French national DESIR cohort

**DOI:** 10.1136/rmdopen-2024-004484

**Published:** 2024-07-23

**Authors:** Anna Molto, Chris Serrand, Sandrine Alonso, Francis Berenbaum, Pascal Claudepierre, Bernard Combe, Laure Gossec, Adeline Ruyssen-Witrand, Alain Saraux, Daniel Wendling, Thierry Lequerre, Maxime Dougados

**Affiliations:** 1 Department of Rheumatology, Assistance Publique - Hôpitaux de Paris, Paris, France; 2 INSERM, Paris, France; 3 Department of Biostatistics, Clinical Epidemiology, Public Health and Innovation in Methodology (BESPIM), CHU Nîmes, Univ Montpellier, Nimes, France; 4 Faculty of Medicine Pierre & Marie Curie Paris VI, Hopital Saint-Antoine, Paris, France; 5 Sorbonne Université, Paris, France; 6 Rheumatology, Hôpital Henri Mondor, Créteil, France; 7 EA EpidermE, Université Paris Est Créteil, Créteil, France; 8 Rheumatology, CHU Montpellier, Montpellier, France; 9 INSERM, Institut Pierre Louis d'Epidémiologie et de Santé Publique, INSERM, Sorbonne Universite, Paris, France; 10 APHP, Rheumatology department, Hopital Universitaire Pitie Salpetriere, Paris, France; 11 Department of Rheumatology, Paul Sabatier University Toulouse III, Toulouse, France; 12 Rheumatology, CHU Brest, Brest, France; 13 Rheumatology, CHU J Minjoz, Besancon, France; 14 Rheumatology Department, Rouen University Hospital, Inserm Unit 905 & Institute for Biomedical Research, University of Rouen, Rouen, France; 15 Hopital Cochin, Rheumatology, Université Paris Descartes Faculté de Médecine, Paris, France

**Keywords:** Axial Spondyloarthritis, Spondylitis, Ankylosing, Epidemiology

## Abstract

**Background:**

Inception cohorts aim to describe chronic diseases from diagnosis and over years of follow-up. Axial spondyloarthritis (axSpA) diagnosis might be challenging during the first years of the disease. Thus, identifying the features that will be associated with a confirmed diagnosis over time is key.

**Objectives:**

To assess the frequency and the predisposing factors for a change of an initial diagnosis in an inception axSpA cohort.

**Methods:**

DESIR is an ongoing national multicentre inception axSpA cohort with currently 12.5 years of follow-up. At the entry visit and confirmed at each visit, the diagnosis of axSpA was based on the opinion of the treating rheumatologist. Follow-up was interrupted in case of a change in this initial diagnosis. Multiple imputation was used to estimate the probability of a change in the initial diagnosis of axSpA for each patient lost to follow-up. Factors predisposing to an unchanged diagnosis of axSpA were then assessed using a multivariate logistic regression model on the imputed data sets.

**Results:**

Of the 708 patients included, over 10 years of follow-up, 45 (6.4%) were excluded due to a diagnosis change and 300 (42.4%) patients were lost to follow-up. Based on the imputation of these 300 patients, a change in their initial axSpA diagnosis was estimated in 42 (14.0%). Factors predisposing to an unchanged initial axSpA diagnosis during follow-up were (ORs (95% CIs)): radiographic sacroiliitis: 17.0 (4.1 to 71.0); psoriasis: 5.3 (2.0 to 14.3); CRP≥6 mg/L: 2.7 (1.3 to 5.3); good NSAID response: 2.5 (1.5 to 4.2); HLA B27+: 2.0 (1.3 to 3.3); anterior chest wall pain: 2.0 (1.2 to 3.3) and female sex: 1.9 (1.2 to 3.0).

**Conclusion:**

These data suggest that a change in diagnosis in recent onset axSpA exists, but is not frequent, and is less likely to occur in the presence of objective features at baseline.

**WHAT IS ALREADY KNOWN ON THIS TOPIC**

- Conventional disease description typically involves cross-sectional studies and retrospective analyses, often conducted in specialised centres, resulting in a bias towards more severe cases. This has led to a portrayal of axial spondyloarthritis (axSpA) as having a severe and poor prognosis, which can be distressing for newly diagnosed patients.

**WHAT THIS STUDY ADDS**

- This study, using data from the DESIR cohort, reveals that the diagnosis of axSpA can change in approximately 10% of cases over 10 years. It also shows that using classification criteria at baseline in such cohorts may exclude many patients, as a significant number will only meet the criteria during follow-up.

**HOW THIS STUDY MIGHT AFFECT RESEARCH, PRACTICE OR POLICY**

- Our findings suggest that including patients based on the treating physician’s diagnosis in inception cohorts, despite its risk of changes, is a more inclusive approach. These data suggest that a change in diagnosis in recent onset axSpA exists, but is not frequent, and is less likely to occur in the presence of objective features at baseline.

## Introduction

 The conventional approach to describing a disease involves assessing the patient’s condition at a specific point in time (eg, cross-sectional studies) and retrospectively examining its clinical features and natural history. However, these studies are typically conducted in specialised centres with expertise in the disease, resulting in a bias of capturing patients with more severe and refractory cases. The findings from these analyses are typically reported in traditional textbooks^1^ and serve as the standard description or presentation of the disease for the medical community, including medical students. In the field of axial spondyloarthritis (axSpA), such studies have portrayed a rather severe and poor prognosis,^2^ and this description can be distressing for patients who have recently received a diagnosis and are concerned about their long-term prognosis.

However, an alternative approach based on data from inception cohorts^3^ is now emerging. Inception cohorts involve the long-term prospective follow-up of patients who have been recently diagnosed with a disease.^4 5^ The advantage of these cohorts is that the data collected over time better reflect the long-term prognosis of patients encountered in everyday clinical practice. For instance, in rheumatoid arthritis, the 10-year outcomes of patients with recent-onset synovitis suggestive of rheumatoid arthritis were found to be quite favourable, with a significant proportion of patients maintaining an acceptable status without any disability.^6^ Such information is reassuring for patients who have recently received a diagnosis.

Some inception cohorts, namely for early RA, have used classification criteria as their inclusion criteria,^7 8^ but more often, patients are rather included according to the diagnosis from the treating rheumatologist.^3 9^ The reason for this lies in the fact that classification criteria are usually very specific at the cost of a lower sensitivity in particular at early stages of the disease. Conversely, the use of the rheumatologist’s diagnosis as entry criteria is considered more sensitive at the cost of a lower specificity. In clinical practice, the initial diagnosis of axSpA has been reported to be challenging and can change after several months or years of follow-up; thus, it seems crucial to be able to identify the baseline features that will be associated with a consistent diagnosis over time.

Given these preliminary considerations, we seized the opportunity provided by the DESIR cohort, which followed patients with recent onset (axial inflammatory symptoms for less than 3 years) axSpA for 10 years, to evaluate (a) the frequency and the baseline predisposing factors of a change in the entry visit initial diagnosis and (b) the impact of conducting a cohort based on classification versus diagnostic criteria in this cohort.

## Patients: methods

### Patients

The DESIR cohort (NCT01648907) is currently ongoing and has been previously described.^10 11^ Briefly, consecutive patients aged 18–50 with inflammatory back pain and a duration of ≥3 months and <3 years were included in 25 centres in France if the treating rheumatologist considered the symptoms highly suggestive of axSpA (a score ≥5 on a scale from 0 to 10, where 0 indicated ‘not suggestive’ and 10 indicated ‘very suggestive’). Between December 2007 and April 2010, 708 patients were included.

The investigators were asked to maintain all enrolled patients in the cohort during the first 2 years of follow-up. Starting at year 2 and during all subsequent visits up to year 10, the investigators had the opportunity to exclude patients from the cohort if they were convinced that another diagnosis than axSpA could explain the symptoms observed.

A detailed description of the study protocol is available on the DESIR website (https://www.lacohortedesir.fr/: desir in English).

The research proposal for this particular analysis was approved in January 2022 by the scientific committee of the DESIR cohort. Patients were not involved in the design, conduct, reporting or dissemination plans of our research.

### Data collected

At baseline, the following variables were collected: demographics (age, gender), socioprofessional status, HLA B27 antigen and symptom duration.

During all visits (semiannually during the first 2 years and annually thereafter), the following variables were collected:

- Items to check if the patients fulfilled the axSpA criteria (Amor^12^ and ASAS^13^).
- Past or present anterior chest wall pain (including date of onset and exact localisation since disease onset).
- Disease activity and severity parameters, including BASDAI (Bath Akylosing Spondylitis Disease Acitivty Index),^14^ ASDAS (Axial Spondyloarthritis Disease Activity Score),^15^ CRP (C-reactive Protein) and BASFI Bath Akylosing Spondylitis Functional Index).^16^
- Impact of the disease on the daily life of the patients, including ASAS HI (ASAS Heath Index)^17^ and SF-36 (Short form Questionnaire 36).^18^
- Extraspinal manifestations of the disease, including synovitis, dactylitis and enthesitis.
- Extramusculoskeletal manifestations of the disease, including psoriasis, uveitis and inflammatory bowel disease.
- Main comorbidities, including severe gastrointestinal events, hypertension, major atherosclerotic cardiovascular events, diabetes, cancer and infection (tuberculosis and other severe infections).
- Pharmacological treatment modalities, including NSAID (Non-steroidal Anti-inflammatory drugs) intake according to the ASAS-NSAID scoring system,^19^ conventional synthetic and targeted DMARDs Disease-modifying antirheumatic drugs).
- Requirement for surgical treatment modalities, including total hip replacement due to coxitis and spinal vertebrotomy.
- Socioprofessional status, including the number of days of sick leave and pension of invalidity,

### Statistical analysis

#### Studied populations

This study includes three populations: ‘confirmed’, referring to patients who had a full 10-year follow-up with no change in diagnosis; ‘other diagnosis’, referring to patients who were excluded from the cohort due to a documented change in diagnosis during the follow-up (after year 2, as per protocol); and ‘lost to follow-up’, referring to patients who were lost at some point during the 10-year follow-up.

It is important to note that some patients classified as ‘lost to follow-up’ may have been lost due to a diagnosis change that could not be documented in the cohort, which may undermine the representativeness of estimates and introduce selection biases when the factors affecting follow-up are associated with outcomes.

To overcome this, multiple imputation by fully conditional specification was performed and the probability of each ‘lost to follow-up’ patient being excluded because of ‘other diagnosis’ was estimated.^20^ Baseline clinical, biological and radiological characteristics, as well as potential changes in baseline diagnosis and the 10-year outcomes, were used to create 10 imputed datasets. To estimate the proportion of lost to follow-up patients who would have been excluded because of a ‘other diagnosis’, we arbitrarily chose to retain patients who were classified as such in at least 7 out of the 10 imputation sets.

Characteristics of the patients at year 10 based on their status (‘confirmed’, ‘lost to follow-up’ and ‘other diagnoses’) are described. Quantitative variables are presented with their mean and SD or median with IQR, and qualitative variables with their frequency and associated proportions.

Furthermore, the probability of being lost to follow-up at each visit was estimated by Kaplan-Meier survival curves.

#### Predisposing factors of a change in the baseline diagnosis of axSpA

In this analysis, the change in baseline diagnosis was defined as the dependent variable, and all parameters collected at baseline were considered independent variables. Variables were analysed through univariate and then multivariate logistic regression on imputed datasets, and the results were aggregated according to Rubin’s rules.^20^ Factors with a p value less than 0.20 in the univariate analysis were retained for the multivariate analysis. In the multivariate analysis, a step-by-step backward selection was performed until only significant factors remained. This process was repeated for the 10 imputed datasets, and variables that remained in at least 8 of the 10 models (and not datasets) were included in the final aggregated model. The ORs with their 95% CIs are presented.

#### Diagnosis versus classification criteria

The number and proportion of patients fulfilling at baseline the following axSpA classification criteria: (Amor^12^ and ASAS^13^ criteria) as well as the number and proportion of patients fulfilling such criteria after 10 years of follow-up were calculated in the ‘confirmed’ population.

All statistical analyses were performed by using SAS enterprise guide V.7.1 software.

## Results

### Patients and study course

**Figure 1:**
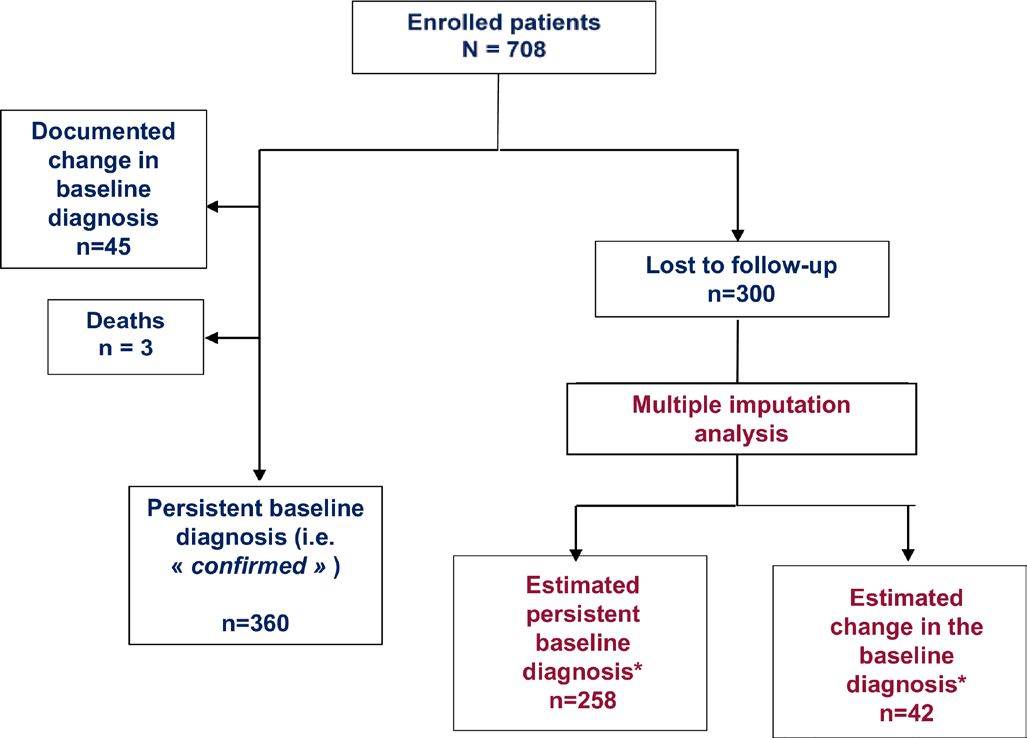
Flow chart of 708 patients with recent low back pain considered as having axSpA, over their 10-year follow-up in the Devenir des Spondyloarthrites Récentes (DESIR) cohort. *See the Methods section. We considered a patient as ‘estimated change in the baseline diagnosis’ in case a change in diagnosis was estimated in at least 7 out of the 10 imputed datasets. axSpA, axial spondyloarthritis.

The flow chart of the patients is summarised in figure 1. Of the 708 enrolled patients, 45 were excluded from the cohort due to a change in the baseline diagnosis by their treating rheumatologist. These 45 patients represent 6.4% of the entire cohort and 12.4% of the number of patients who reached the 10-year follow-up (ie, ‘confirmed’ patients). The diagnoses justifying the exclusion of the patients from the cohort were as follows: mechanical back pain (n=30), fibromyalgia (n=13), a non-axSpA undifferentiated inflammatory rheumatic disease (n=1) and no information (n=1). During the 10-year follow-up period, three patients died due to the following causes: suicide (n=1), colorectal cancer (n=1) and cardiac arrest (n=1).

Of the remaining 660 patients, 300 were lost to follow-up during the 10 years with the following estimations: 14.8% (95% CI 12.0% to 17.4%), 25.5% (95% CI 24.0% to 30.8%) and 45.7% (95% CI 41.8% to 49.4%) at the 2-year, 5-year and 10-year visits, respectively (online supplemental figure 1).

In at least 7 (out of 10) multiple imputation data sets, 42 patients (5.9% of the entire cohort and 14.0% of the group of patients lost to follow-up) were imputed as ‘other diagnosis’ (eg, suspected of a change in the axSpA baseline diagnosis); considering these imputations, in total 87 patients (45 documented changes in diagnosis and 42 estimated, according to the multiple imputation results) (ie, 12.2% of the whole cohort) were estimated to be suffering from a disease different than axSpA (online supplemental figure 2).

The baseline characteristics of the patients regarding these different populations are summarised in table 1.

**Table 1:** Baseline characteristics of the patients with regard to their status during the 10-year follow-up period

Baseline characteristics	Completers	Lost to follow-up	Documented change in the baseline diagnosis	Whole population
	n=360	n=300	n=45	n=708
Age (years)*	34±9	32±8	36±8	34±9
Female	192 (53%)	163 (54%)	24 (53%)	381 (54%)
Ethnicity (white caucasian)	328 (91%)	266 (89%)	44 (98%)	641 (91%)
Educational level: university	231 (64%)	159 (54%)	28 (62%)	418 (59%)
Time from first symptoms to baseline (years)	1.5±0.9	1.6±0.9	1.5±0.9	1.5±0.9
HLA B27: positive	231 (64%)	160 (54%)	18 (40%)	410 (58%)
Extraspinal manifestations (past or present)				
Enthesitis	177 (49%)	147 (49%)	22 (49%)	348 (49%)
Synovitis	85 (24%)	62 (21%)	2 (4%)	151 (21%)
Dactylitis	50 (14 %)	39 (13%)	1 (2 %)	92 (13%)
Extramusculoskeletal manifestations (past or present)				
Psoriasis	67 (19%)	48 (16%)	3 (7%)	119 (17%)
Uveitis	35 (10%)	23 (8%)	1 (2%)	60 (8%)
IBD	17 (5 %)	11 (4%)	0 (0 %)	28 (4%)
Disease activity				
BASDAI (0–100)	43±21	46±20	50±16	45±20
ASDAS	2.6±1.0	2.7±1.0	2.6±0.6	2.7±0.9
CRP (mg/L)	8.6±14.0	7.7±13.0	2.3±2.5	7.9±13.5
MRI-SIJ inflammation†	143 (41%)	86 (30%)	2 (5%)	231 (34%)
X-rays SIJ structural damage‡	112 (32%)	72 (25%)	2 (4%)	187 (27%)
Quality of life/impact				
BASFI	29.4±23.1	31.4±22.6	32.1±21.4	30.5±22.8
SF-36 Physical score	39.9±9.6	38.7±9.2	37.2±9.9	39.2±9.5
SF-36 Mental score	41.4±11.1	38.9±11.2	39.1±12.3	40.2±11.3
PASS	156 (44%)	117 (39%)	15 (33%)	290 (41%)

* Values given are mean±standard deviationSD for continuous variables and numbers (percentage) for binary variables,.

† MRI-SIJ-inflammation=presence of inflammation at the acroiliac level on MRI based on the opinion of the local reader (either radiologist or rheumatologist), .

‡ X-Rrays-SIJ structural damage=presence of structural damage suggestive of axSpA on pelvic X-Rrays based on the local reader (either radiologist or rheumatology), .

ASDASAxial Spondyloarthritis Disease activity scoreBASDAIBath Akylosing Spondylitis Disease Activity Index BASFIBath Akylosing Spondylitis Functional Index CRPC-reactive proteinHLAB27Human Leucocyte Antigen 27IBDinflammatory bowel diseasePASSpatient acceptable symptom stateSF-36Short Form questionnaire 36SIJSacroiliac joints

### Predisposing factors of a confirmed baseline diagnosis

Factors predisposing to an unchanged initial axSpA diagnosis during follow-up were (ORs (95% CIs)): radiographic sacroiliitis: 17.0 (4.1 to 71.0); psoriasis: 5.3 (2.0 to 14.3); CRP≥6 mg/L: 2.7 (1.3 to 5.3); good NSAID response: 2.5 (1.5 to 4.2); HLA B27+: 2.0 (1.3 to 3.3); anterior chest wall pain: 2.0 (1.2 to 3.3) and female sex: 1.9 (1.2 to 3.0) (table 2).

**Table 2:** Baseline predisposing factors of a maintained (no change) diagnosis of axSpA during the 10-year follow-up period (results of the multivariate analysis on the imputed datasets)

Baseline characteristics	Change in the diagnosis during the 10-year period		OR (95% CI)
	Yes*	No*	
	14.8 (14.5–14.8)	85.2 (85.2–85.5)	
X-rays SIJ† structural damage	4.8 (3.8–4.8)	30.5 (30.3–31.1)	16.97 (4.06 to 71.02)
Psoriasis (past or present)	4.8 (3.9–4.9)	18.9 (18.8–19)	5.33 (1.99 to 14.27)
CRP≥6 mg/L	11 (10.1–11.1)	33 (32.9–33.1)	2.65 (1.33 to 5.88)
Good NSAID response	62.9 (62.1–63.1)	83.0 (82.8–83.1)	2.50 (1.51 to 4.15)
HLA B27 positive	40.0 (39.0–40.8)	61.2 (61–61.2)	2.04 (1.27 to 3.27)
Anterior chest wall pain	31.4 (30.8–31.4)	46.9 (46.9–47)	2.02 (1.23 to 3.32)
Female	46.7 (46.2–47.6)	55.1 (54.9–55.1)	1.88 (1.17 to 3.01)

* Data provided are the observed median, minimal and maximal percentage values from the imputed datasets.

† X-Rrays SIJ structural damage=presence of structural damage suggestive of axSpA on pelvic X-Rrays based on the local reader (either radiologist or rheumatologist).

### Diagnosis versus classification as entry criteria

The fulfilment of axSpA classification criteria (spondyloarthritis., Amor and ASAS criteria) regarding the patients’ status during the 10-year follow-up period was evaluated: 21 (49%) and 16 (36%) out of the 45 patients with a documented change in diagnosis fulfilled the baseline Amor and ASAS criteria, respectively. The proportion of patients fulfilling these criteria in the ‘confirmed’ population went from 85% to 100% for the Amor criteria and from 68% to 83% for the ASAS criteria from baseline to year 10, respectively.

## Discussion

These results provide insights into the pros and cons of selecting inclusion criteria in an inception cohort. Based on the diagnosis by the treating physician, there was a risk of changes in the baseline diagnosis over time, which occurred in approximately 10%–15% of our cohort. Interestingly, when examining the classification criteria, we observed the following: first, that a significant proportion of patients whose treating physician changed the diagnosis over time did actually meet the classification criteria at baseline (47% and 36% for the Amor and ASAS criteria, respectively); and second, that the percentage of patients meeting the classification criteria increased during the 10-year follow-up period, suggesting that enrolling patients based on the fulfilment of classification criteria would have excluded a significant number of patients. These data confirm that the inclusion of patients in an inception cohort should be based on the diagnosis by the treating physician, but the risk of changes in the baseline diagnosis must be considered in subsequent analyses and sample size estimations. Furthermore, and from a more clinical point of view, these findings highlight that despite it has been suggested that axSpA diagnosis can be challenging, in our cohort the final proportion of persistent diagnosis over time was very high (ie, more than 85%).

Also, during the conduct of these analyses, we encountered the issue of missing data due to patients being lost to follow-up during the study period. To address this, we chose to use multiple imputations. This technique has the advantage of using the full extent of available information and allows to provide unbiased estimates under the assumption of data missing at random.^21 22^ However, since the results obtained are estimations based on observed data, they may differ from reality. Moreover, the high number of missing data and the rarity of some events prevented us from applying this approach to a few outcomes, particularly those related to disease management during the study period. Nonetheless, this imputation method is less prone to bias than complete-case analysis.^23^

Not surprisingly, baseline characteristics associated with a change in the baseline diagnosis mirrored the parameters that have been reported to have a higher likelihood ratio for diagnosis^24^: B27 negativity, the absence of radiographic-structural damage, poor or no response to NSAIDs, the absence of psoriasis and the absence of anterior chest-wall pain. More intriguing and unexpected were the association of the female gender with an unchanged baseline diagnosis.

In summary, these data suggest that a change in diagnosis at the inclusion visit and the risk of follow-up should be considered in inception cohorts but that statistical models including multiple imputations could facilitate assessment of the long-term prognosis of the disease despite these challenges.

## supplementary material

10.1136/rmdopen-2024-004484 online supplemental file 1

## Data Availability

Data are available on reasonable request.
