# Supplementary material for: Diagnosis challenges in inception cohorts in axial spondyloarthritis: the case of the French national DESIR cohort
Source: RMD Open. 2024 Jul 23;10(3):e004484. doi: 10.1136/rmdopen-2024-004484 (PMC11337680; doi:10.1136/rmdopen-2024-004484)
Supplement: online supplemental file 1 [file rmdopen-10-3-s001.pdf]

Supplementary data:

Supplementary figure 1: Kaplan-Meier curve representing the 10-year time to loss of follow-up in the DESIR cohort

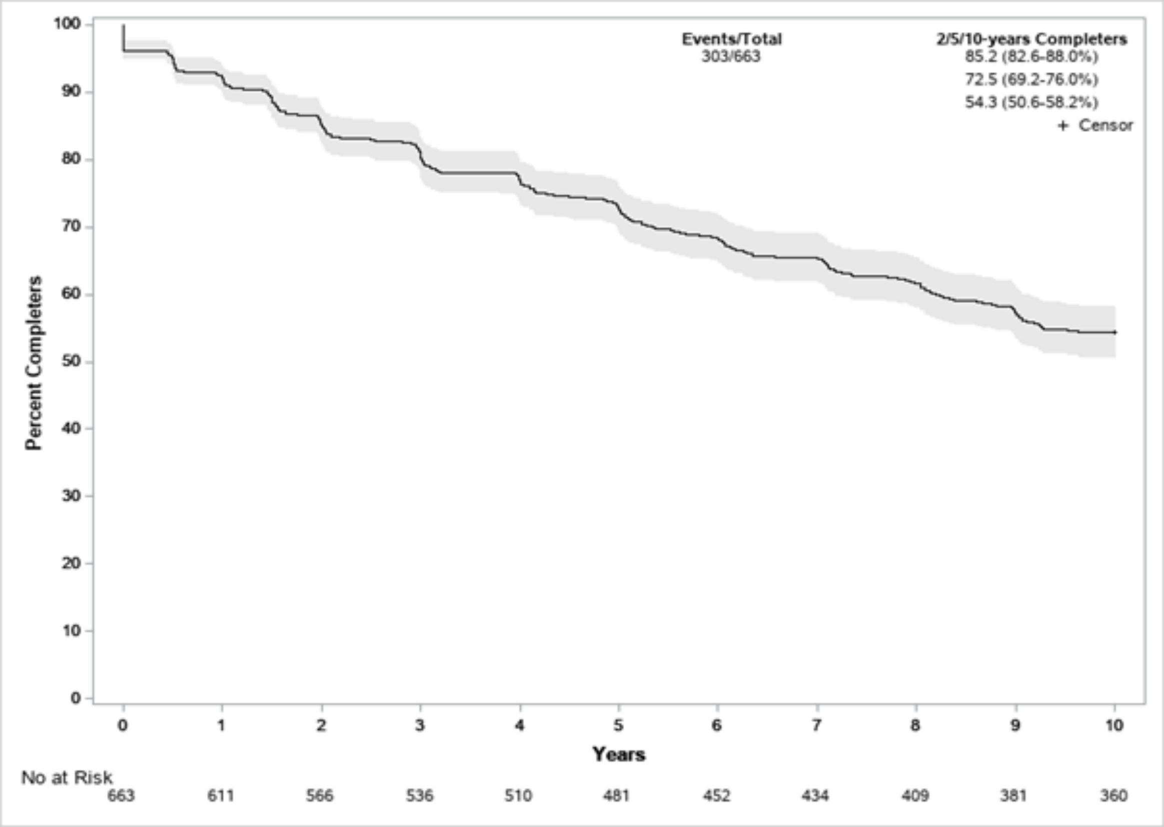

Supplementary figure 2: results of the 10 imputation data sets exploring the probability of a change in the original diagnosis at the time the patients were lost of follow-up

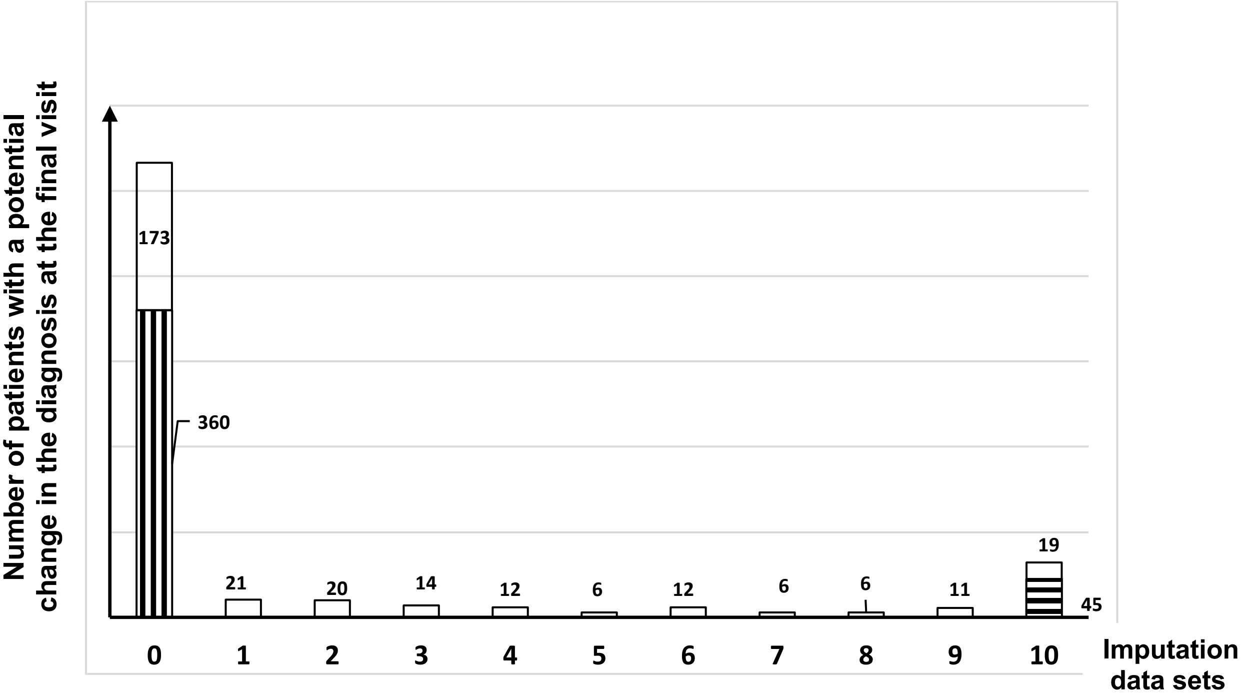

= Patients who completed the 10 year period with a maintained diagnosis of axSpA (n = 360)

= Patients excluded from the cohort because of a documented change in the baseline diagnosis of axSpA during the 10-year period (n =45)

= Patients from the « lost of follow-up » group with a suspected change in the baseline diagnosis of axSpA during the 10-year period (173 patients in none of datasets to 19 in the 10 data sets)
